# The Effectiveness of Liquid-Phase Microextraction of Beta-Blockers from Aqueous Matrices for Their Analysis by Chromatographic Techniques

**DOI:** 10.3390/molecules30051016

**Published:** 2025-02-22

**Authors:** Mihail Simion Beldean-Galea, Mihaela-Cătălina Herghelegiu, Vlad-Alexandru Pănescu, Jérôme Vial, Maria Concetta Bruzzoniti, Maria-Virginia Coman

**Affiliations:** 1 Faculty of Environmental Science and Engineering, Babeș-Bolyai University, 30 Fântânele Str., RO-400294 Cluj-Napoca, Romania; vlad.panescu@ubbcluj.ro; 2 “Raluca Ripan” Institute for Research in Chemistry, Babeş-Bolyai University, 30 Fântânele Str., RO-400294 Cluj-Napoca, Romania; virginia.coman@ubbcluj.ro; 3 Chemistry, Biology and Innovation Department, École Supérieure de Physique et de Chimie Industrielles ESPCI Paris PSL, 10 Rue Vauquelin, 75005 Paris, France; 4 Department of Chemistry, University of Turin, Via P. Giuria 5, 10125 Turin, Italy; mariaconcetta.bruzzoniti@unito.it

**Keywords:** DLLME, SFOME, beta-blockers, chromatographic techniques, wastewater samples

## Abstract

Beta-blockers are pharmaceuticals used to treat cardiovascular diseases such as hypertension, angina pectoris, and arrhythmia. Due to high consumption, they are continuously released into the environment, being detected in many aqueous matrices. The aim of this research is to test the effectiveness of two green liquid-phase microextraction procedures, such as dispersive liquid–liquid microextraction (DLLME) and solidification of floating organic droplet microextraction (SFOME) for the selective extraction of eight beta-blockers (atenolol, nadolol, pindolol, acebutolol, metoprolol, bisoprolol, propranolol, and betaxolol) from aqueous matrices for their analysis by gas chromatography (GC) or liquid chromatography (LC). The influence of extraction parameters, such as the type and volume of extraction and disperser solvents, and ionic strength were studied. The developed extraction procedures provide a good enrichment factor for six compounds (61.22–243.97), good extraction recovery (53.04–92.1%), and good sample cleaning for both extraction procedures. Good limits of detection (0.13 to 0.69 µg/mL for GC and 0.07 to 0.15 µg/mL for HPLC) and limits of quantification (0.39 to 2.10 µg/mL for GC and 0.20 to 0.45 µg/mL for LC) were obtained. The developed procedures were successfully applied to the analysis of selected beta-blockers in wastewater samples, proving their applicability to the real samples.

## 1. Introduction

Beta-blockers are widely prescribed pharmaceuticals for the treatment of cardiovascular diseases [1,2]. Cardiovascular diseases are a group of diseases of the heart and blood vessels, such as hypertension, angina pectoris, myocardial infarction, heart failure, and arrhythmia, being a common cause of mortality in all countries [3,4]. These pharmaceuticals block beta-adrenergic receptors, preventing the binding of adrenaline and noradrenaline, which leads to a decrease in heart rate, cardiac output, and implicitly, a diminish in blood pressure [5]. In addition to their beneficial effects on humans, beta-blockers are illegally used in horse racing [6] and in sports such as archery, billiards, and golf, being banned by the World Anti-Doping Agency [5,7,8]. They are also used to prevent stress and death in animals during transport to slaughterhouses [1]. The consumption of beta-blocker drugs in Romania has increased in the last 20 years [9]. The most commonly used beta-blockers for the treatment of cardiovascular diseases are propranolol, metoprolol, sotalol, atenolol, and bisoprolol [1]. Following excretion, beta-blockers are removed into the sewage system from where they reach the wastewater treatment plant and then proceed into the aquatic environment [10]. Iancu et al. [11] found the following concentrations of beta-blockers in wastewater from Romania from the influent: atenolol 29–623 ng/L, bisoprolol 5.1–309 ng/L, betaxolol 6.2–144 ng/L, propranolol 7.0–52.5 ng/L and from the effluent: atenolol 17–300 ng/L, bisoprolol 2.8–170 ng/L, betaxolol 3.1–40 ng/L, propranolol 5.1–41 ng/L. Alder et al. [12] found concentrations of atenolol between LOQ–83 ± 27 ng/L, sotalol between LOQ–52 ± 13 ng/L, metoprolol between LOQ–36 ± 13 ng/L, and propranolol between LOQ–8 ± 1 ng/L in river water from Switzerland. Concerns about the presence of these pharmaceuticals in the aquatic environment are growing due to their harmful effects on organisms [4,10,13] and their belonging to the class of Endocrine Disrupting Compounds, since they can disrupt testosterone levels in male organisms [7].

The presence of beta-blockers at trace concentration levels in aquatic environments requires the development of sensitive, reliable, and rapid methods for their detection. The most used method for the extraction of these pharmaceuticals from aqueous matrices involves solid phase extraction (SPE) [1,8,11,14]. However, the practicality of the SPE method is overshadowed by the use of a large amount of organic solvent and sample, the fact that it generates waste, the extraction cartridge being single use, it is expensive and time-consuming, and that the final extract must be concentrated to obtain a high enrichment factor. To overcome these problems, in recent years, miniaturized techniques have been developed to replace classical extraction techniques. Among these microextraction techniques are solid-phase microextraction (SPME) [15,16], stir-bar sorptive extraction (SBSE) [17,18], fabric-phase sorbent extraction (FPSE) [19,20], µQuEChERS (micro, quick, easy, cheap, effective, rugged, and safe) [21,22], single-drop microextraction (SDME) [23,24], hollow-fiber liquid-phase microextraction (HF-LPME) [25,26], dispersive liquid–liquid microextraction (DLLME) [27], and solidification of floating organic droplet microextraction (SFOME) [28,29]. Dispersive liquid–liquid microextraction has attracted considerable attention due to its advantages, which include low solvent and sample consumption, minimal waste production, cost-effectiveness, simplicity, rapidity, and high enrichment factors [2,30].

DLLME is a tertiary extraction system that consists of the liquid sample, the extraction solvent, and a cosolvent (dispersant or dispersion solvent), which plays a role in facilitating the dispersion of microdroplets of extraction solvent in the aqueous sample [31]. The appropriate mixture of extraction solvent and dispersant is introduced into the aqueous sample and stirred, resulting in the dispersion of small droplets of extraction solvent in the sample, which increases the contact surface between the aqueous sample and the extraction solvent, reducing the extraction time and increasing the enrichment factors [28,29]. The mixture is centrifuged, and the compounds of interest are found in the sedimented phase [31] (in the case of organic solvents that are heavier than water, such as in chloroform or dichloromethane [3]) or in the upper part for solvents lighter than water [32]. In the case of using 1-undecanol or 2-dodecanol, after centrifugation, the sample is placed in an ice-water bath to solidify the extraction solvent which is then collected, melted at room temperature, and analyzed by various chromatographic techniques [29]. The extraction performance is influenced by the type of extraction solvents and dispersants, their volumes [30], and other experimental conditions, such as sample pH or ionic strength [33], requiring different optimization procedures.

Despite all the advantages offered by these miniaturized extraction techniques, the use of SFOME or DLLME for the extraction of beta-blockers from water samples is scarce [27,29]. Generally, these extraction methods are applied to biological samples [3,5,23,34,35].

To fill these gaps, the present research aims to test the effectiveness of two green dispersive liquid–liquid microextraction procedures (DLLME and SFOME) for the selective extractions of eight beta-blockers (atenolol, nadolol, pindolol, acebutolol, metoprolol, bisoprolol, propranolol, and betaxolol) from aqueous matrices for their analysis by liquid chromatography or gas chromatography, the most widely used analytical methods for the analysis of the selected compounds. Moreover, the developed DLLME-GC-MS and SFOME-LC-PDA methods were then successfully applied to the analysis of these drugs in wastewater samples to verify their applicability to real samples.

## 2. Results

### 2.1. Optimization of DLLME and SFOME Conditions

For the optimization of DLLME and SFOME procedures we considered our previous works [33,36,37,38] in which we used 1-undecanol and chloroform as the most suitable solvents for extraction and acetonitrile as the dispersion solvent.

For a better comparison of the extraction method performance, we used the same protocol for both SFOME and DLLME. The protocol used is as follows: A volume of 10 mL of distilled water alkalinized to pH 11 with a NaOH solution was placed in a 15 mL polypropylene conical tube, and then the water sample was spiked with 1000 ng of each pharmaceutical product. The obtained sample was then optimized for the extraction protocol under different extraction conditions using a 2^3^ full factorial experimental design. The relative extraction recovery (ER) of the studied pharmaceutical products from each experiment was used to express the extraction efficiency and to optimize the extraction conditions.

For both the DLLME and SFOME protocols, a total of eight experiments (2^3^) were performed using different values of the three variables (volume of extraction solvent (*X*1) and dispersant (*X*2) and amount of salt (*X*3)) at minimum (−1), maximum (+1), and three experiments at the medium setpoint (0), respectively. Tables S1 and S2 of the Supplementary Materials present all the extraction recovery data obtained for each experiment performed. Table S3 provides the experimental conditions of each protocol used in experiments 1 to 11. Each data set (for DLLME and SFOME) was used to simultaneously optimize the extraction conditions using desirability functions (Supplementary Materials, Figures S1 and S2).

The relationship between response *Y* (extraction recovery) and the three independent variables—*X*1, *X*2, and *X*3—can be mathematical modeled by a linear polynomial equation [39], as follows:

(1) $$Y=\beta_{0}+\beta_{1}X1+\beta_{2}X2+\beta_{3}X3+\beta_{12}X1X2+\beta_{13}X1X3+\beta_{23}X2X3\;+\beta_{123}X1X2X3$$

where $\beta_{0}$ is a constant; $\beta_{1}$, $\beta_{2}$, and $\beta_{3}$ are linear coefficients; and $\beta_{12}$, $\beta_{13}$, $\beta_{23}$, and $\beta_{123}$ are interaction coefficients.

The values of the coefficients of the polynomial equation can have positive values indicating a positive effect on the extraction recovery or negative values indicating a negative effect. The magnitude of these effects can be observed in Supplementary Tables S4 and S5, which include the significance levels of the factors and their interactions both for DLLME and SFOME.

For SFOME, the predicted optimal conditions have the following coded values: +1 for the dispersant volume, +1 for the salt amount, and +1 for the extraction solvent volume (Supplementary Materials Figure S1). These coded values correspond to the following experimental conditions: salt amount (NaCl): 2 g, dispersant volume (acetonitrile): 250 µL, and extraction solvent volume (1-undecanol): 100 µL.

For DLLME, the optimal conditions predicted by the model were in coded values: +1 for the dispersant volume, +1 for the salt amount, and 0 for the extraction solvent volume (Supplementary Materials Figure S2), which correspond to the following experimental conditions: salt amount (NaCl): 2 g, dispersant volume (acetonitrile): 250 µL, and extraction solvent volume (chloroform): 75 µL.

A summary of the maximum, minimum, and optimal extraction recoveries obtained by simulation for each compound in SFOME and DLLME is presented in Table 1.

Since the conditions predicted by the model are optimal theoretical conditions for their use in practical experiments, it is necessary that they be experimentally validated.

### 2.2. Validation of SFOME-LC-PDA Method

The DLLME-SFO-LC-PDA method was validated in terms of linearity, sensitivity (LOD and LOQ), intra-day precision (repeatability, %RSD) and inter-day precision (%RSD), and accuracy (recovery) in accordance with the International Committee for Harmonization (ICH) criteria [40].

The linearity of the method was evaluated based on the calibration curve created by plotting the peak area of the analyte versus the analyte concentration. For this, three replicates of different standard mixtures made by successive dilutions of the stock solution were injected in the range of 1.56 and 50 µg/mL of each compound. Good linearities were obtained for all analytes, with correlation coefficients (R^2^) greater than 0.995 (Table 2).

The *sensitivity* was determined in terms of limit of detection (LOD) and limit of quantification (LOQ) by statistical approaches using standard deviation (SD) of the regression line and the slope (S) each the calibration curve, using the following mathematical formulas: LOD = 3.3 × SD/S, LOQ = 10 × SD/S, according to ICH [40].

The instrument limits of detection for the beta-blockers were between 0.07 and 0.13 µg/mL and the instrument limits of quantification were between 0.20 and 0.45 µg/mL (Table 2).

The *accuracy* was expressed by the extraction recovery of analytes from the spiked water sample. The optimal SFOME parameters predicted by the model were used. Thus, a volume of 10 mL of Milli-Q water, alkalinized to pH 11 with a solution of 0.1 molar of NaOH, was spiked with 1000 ng of each selected compound. After adding 2 g of NaCl, a mixture containing 100 µL of 1-undecanol as an extraction solvent, and 250 µL acetonitrile as a disperser was added to the samples, followed by centrifugation for 5 min at 5000 rpm.

The obtained results were between 59.99 and 87.55% for all studied pharmaceuticals, except for atenolol and nadolol, for which the recoveries were 14.71 and 29.85%, respectively (Table 2).

The *intra*- and *inter-day precision* were evaluated by relative standard deviations (RSD%) on Milli-Q water samples spiked with 1000 ng of each pharmaceutical. The obtained RSD% values were below 4.87% for the intra-day precision and below 6.38% for the inter-day precision for all the pharmaceuticals tested (Table 2), thus agreeing with the requirements of the method validation procedures for compounds in the µg/mL concentration range.

The *enrichment factors* were calculated (Equation (2)) based on the ratio of analyte concentration in the collected organic phase and the initial concentration of the analyte in the liquid sample. The results showed that the developed SFOME protocol provided an EF between 61.22 and 89.34, except for atenolol and nadolol, for which the EF was 15.01 and 30.46, respectively (Table 2), higher than the EFs obtained by [23,29,35].

### 2.3. Validation of DLLME-GC-MS Method

The DLLME-GC-MS method was also validated according to ICH [40]. Linearity of the method was tested for the concentrations of the studied pharmaceuticals between 1.25 µg/mL and 100 µg/mL. The method has a good linearity with R over 0.998, low LODs and LOQs in the range of µg/mL, but higher relative standard deviations (RSD) (6.47–12.60%) for intra- and inter-day precision compared with SFOME-LC-PDA method (Table 3).

The extraction recovery range between 53.04 and 92.1% for all studied pharmaceuticals, except for atenolol and nadolol, for which the recoveries were <LOQ and 3.14%, respectively, while the enrichment factors (EF) were between 198.89 and 243.97, except for atenolol and nadolol, for which the EF was not estimated and 11.79, respectively (Table 3). The higher EF obtained in DLLME compared to SFOME is due to the fact that the volume of extraction solvent collected in DLLME was 40 µL compared to 75 µL in SFOME.

### 2.4. Analysis of Wastewater

The applicability of the proposed SFOME and DLLME protocols for the analysis of selected beta-blockers in wastewater was tested. For this purpose, 10 mL of wastewater was spiked with 625 ng of selected compounds and subjected to the extraction protocols described above. Before extraction, the wastewater samples were filtered with a 0.45 µm nylon white membrane filter to remove suspended particles.

Three experiments were performed for each extraction protocol, and subsequently, the enrichment factors and extraction recoveries were calculated. No significant interferences were observed in wastewater samples, indicating that the optimized sample preparation can eliminate most of the matrix components.

**Figure 1:**
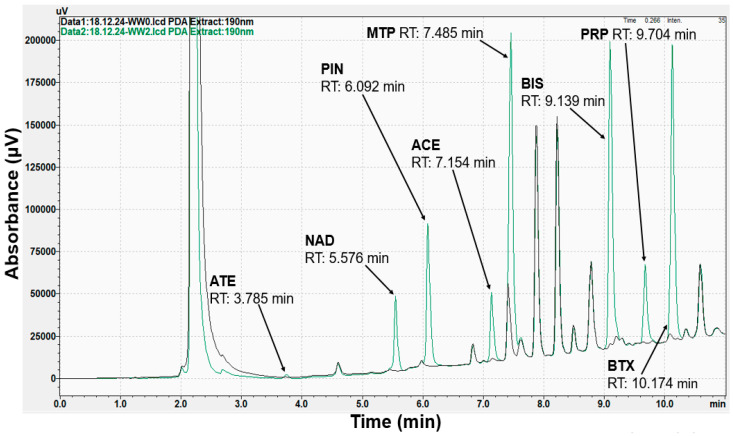
HPLC-PDA chromatograms obtained at 190 nm of wastewater (black color) and spiked wastewater samples (green color); ATE–atenolol, NAD–nadolol, PIN–pindolol, ACE–acebutolol, MTP–metoprolol, BIS–bisoprolol, PRP–propranolol, BTX–betaxolol.

**Figure 2:**
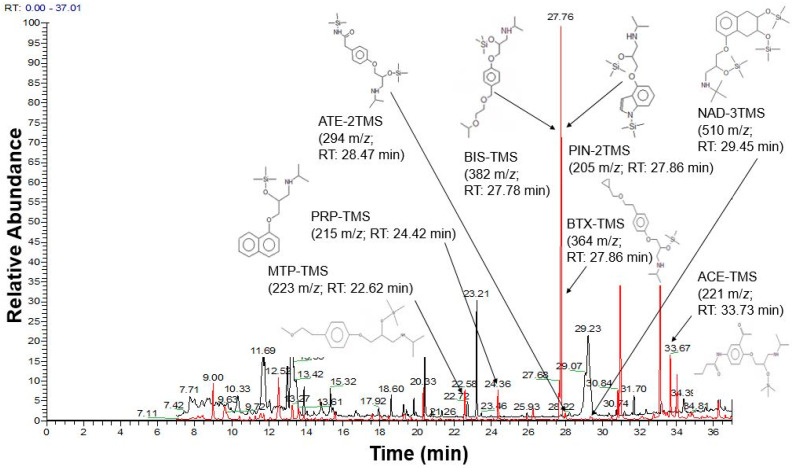
Total ion GC chromatograms of wastewater (black color) and spiked wastewater sample (red color); ATE–TMS–atenolol–TMS, NAD–TMS–nadolol–TMS, PIN–TMS–pindolol–TMS, ACE–TMS–acebutolol–TMS, MTP–TMS–metoprolol–TMS, BIS–TMS–bisoprolol–TMS, PRP–TMS–propranolol, BTX–TMS–betaxolol–TMS.

The HPLC-PDA chromatograms related to the non-spiked and spiked wastewater sample are shown in Figure 1 and the GC-MS chromatograms in Figure 2.

The results obtained for SFOME-LC-PDA of wastewater (Table 4) showed reasonable extraction recovery values ranging from 58.94 to 86.40%, except for atenolol (1.58%) and nadolol (19.20%).

For DLLME-GC-MS the results obtained on wastewater show extraction recovery values ranging from 63.34 to 82.13%. Atenolol was not detected, while for nadolol the recovery was 3.95% (Table 4).

By comparing the results obtained for SFOME-LC-PDA and DLLME-GC-MS of wastewater it can be seen that both extraction methods give adequate extraction for six of the eight selected beta-blockers. For atenolol and nadolol, none of the proposed protocols provide good extraction recoveries.

When comparing GC-MS and LC-PDA chromatographic methods, each method offers some advantages and disadvantages that are important to consider.

While GC-MS offers high specificity by using MS in compound identification and uses gases as a mobile phase that does not produce waste, this method is limited to the analysis of compounds with low boiling points and which are stable under thermal treatments. For compounds with high boiling points or unstable under thermal treatments, such as beta-blockers, the analysis involves an additional derivatization step that often requires reagents with high toxicity and can introduce additional errors in the quantification of target compounds. This can be seen in Table 3, where the precision has higher RSD% values compared to the LC method.

In contrast, the LC-PDA method allows the analysis of compounds in a wide range of polarities and offers the advantage of analyzing the extract as it results from the extraction, without any processing, but the method produces waste from the analysis consisting of the mobile phases used and has a lower specificity compared to GC-MS.

However, if we consider that the PDA detector can provide additional compound identification using UV spectra at different wavelengths, we can assume that this method offers better conditions for the analysis of selected compounds and, at the same time, a more environmentally friendly alternative in terms of sample processing.

### 2.5. Comparison with Other Studies

The SFOME and DLLME performances obtained in this study were compared with other sample preparation approaches for the microextraction of beta-blockers and are given in Table 5. The performances were evaluated in terms of sample volume, volume of extraction solvent, LOQ, and ER.

As can be observed in Table 5, our developed extraction protocol can provide satisfactory performance, comparable to others used for the microextractions of beta-blockers from different matrices. It is worth noting that, in general, these microextractions are applied to a small number of compounds (maximum five [3]), while in our study eight pharmaceutical compounds were analyzed simultaneously. Since our extraction method has a low LOQ, a small amount of extraction solvent, and a good recovery of over 58.94%, we can affirm that the proposed protocols fulfill the requirements for a green method of extraction.

## 3. Materials and Methods

### 3.1. Chemicals and Reagents

The reference substances of the studied pharmaceuticals (atenolol, nadolol, pindolol, acebutolol, metoprolol, bisoprolol, propranolol, and betaxolol) were purchased from Sigma-Aldrich (Steinheim, Germany). The molecular structure and some physicochemical properties of the studied pharmaceuticals are presented in Supplementary Materials (Table S6).

Stock standard solutions of individual compounds (nadolol, pindolol, acebutolol, metoprolol, propranolol, and betaxolol), at a concentration of 1000 µg/mL each, were prepared in methanol, while solutions of atenolol and bisoprolol were prepared in acetonitrile.

All prepared stock standards were stored at 4 °C in the dark until analysis. Acetonitrile and methanol of HPLC grade, 1-undecanol, sodium chloride, monopotassium phosphate, sodium hydroxide, chloroform, and *n*-hexane were all purchased from Merck (Darmstadt, Germany). The derivatization agent N,O-bis(trimethylsilyl)trifluoroacetamide (BSTFA) with 1% trimethylchlorosilane (TMCS) was purchased from Cerilliant (Round Rock, TX, USA).

Milli-Q water was prepared using a Milli-Q-Plus ultrapure water system (Millipore, Milford, MA, USA). For the centrifugation of the samples, an Eppendorf centrifuge, model 5804 R (Eppendorf, Wien, Austria) was used, and a lab oven Memmert UFE 400 (Memmert, Schwabach, Germany) was used for the derivatization procedure.

### 3.2. Instrumentation

HPLC analyses were carried out using Shimadzu equipment (SLC-40D) with a photodiode detector (SPD-M40). LabSolution software (Version 5.101) was used for the control of the instrument and data acquisition. The separation of the studied pharmaceuticals was performed on a Kinetex C18 (250 × 4.6 mm, 5 µm; Phenomenex, Torrance, CA, USA) column with a flow rate of 1.0 mL/min and an oven temperature of 40 °C. The injection volume was 20 µL. The gradient elution program (acetonitrile (ACN) and KH_2_PO_4_ 25 mM) was used starting at 10% ACN, increasing to 40% ACN for 8 min, increasing to 50% ACN for 2 min, and then a 1 min increase to 80% ACN, and held for 7 min, with an equilibrium time between analyses of 8 min. The analysis time is 18 min. The specific UV wavelengths used for PDA detection were as follows: atenolol, nadolol, metoprolol, bisoprolol, betaxolol—190 nm; pindolol—205 nm; acebutolol—231 nm; propranolol—223 nm.

GC–MS analyses were carried out using a Focus GC equipment with a DSQ II mass spectrometer (single quadrupole) controlled by XCalibur software (v3.0.63, Thermo Electron Corp.) and TriPlus Autosampler (Thermo Electron Corporation, Waltham, MA, USA). The separation of the studied pharmaceuticals was performed on HP-5MS (30 m × 0.25 mm, 0.25 µm; Thermo Fisher Scientific, Norristown, PA, USA) column; the carrier gas was Helium (purity 99.999%) at a flow rate of 1 mL/min. The column temperature was initially set at 100 °C, and then raised by 5 °C/min to 250 °C, and from 250 °C to 300 °C by increments of 10 °C/min. The GC inlet temperature was 310 °C and the transfer line temperature was kept at 300 °C. The ionization was performed in the electron impact mode (70 eV, ion source temperature 220 °C) and the acquisition of mass spectra was performed by scanning from 100 to 550 m/z to obtain the mass spectra of the studied pharmaceuticals. The quantification was performed by selected ion monitoring (SIM) mode and comparison of relative retention times (RT): metoprolol (223 m/z, RT: 22.64 min), propranolol (215 m/z, RT: 24.43 min), pindolol (205 m/z, RT: 27.86 min), bisoprolol (382 m/z, RT: 27.78 min), betaxolol (364 m/z, RT: 27.86 min), atenolol (294 m/z, RT: 28.47 min), nadolol (510 m/z, RT: 29.45 min), acebutolol (221 m/z, RT: 33.73 min).

Due to their polarity, our pharmaceuticals were analyzed as derivative compounds. The derivatization protocol was performed with 100 µL BSTFA with 1% TMCS, for 1 h at 100 °C, according to our previous study [41].

### 3.3. Extraction by SFOME and DLLME Protocol

The SFOME extraction protocol was performed as follows: A volume of 10 mL of distilled water, alkalized to pH 11, was introduced into a 15 mL polypropylene centrifuge tube. Each sample was spiked with 1000 ng of each drug (ATE, PIN, NAD, ACE, PRP, BTX, MTP), to which 2 g of NaCl were added, followed by vigorous shaking of the mixture until the salt dissolved. A total of 350 µL of a mixture consisting of 100 µL 1-undecanol and 250 µL acetonitrile were added, shaken, and then centrifuged for 5 min at 4000 rpm. After centrifugation to separate the two phases, the polypropylene tube was cooled in an ice-water bath for 15 min to solidify the 1-undecanol. The 1-undecanol was collected with a spatula and transferred to a conical flask. After melting the extract at room temperature, a volume of 20 µL was directly injected into the HPLC-PDA for analysis.

The DLLME extraction protocol was performed as follows: a volume of 10 mL of distilled water, alkalized to pH 11, was introduced into a 15 mL polypropylene centrifuge tube. Each sample received 1000 ng of each drug (ATE, PIN, NAD, ACE, PRP, BTX, MTP), to which 2 g of NaCl was added, followed by vigorous stirring of the mixture until the salt dissolved. Then, 325 µL of a mixture consisting of 75 µL chloroform and 250 µL acetonitrile was added, stirred, and then centrifuged for 5 min at 4000 rpm. After centrifugation, a drop of chloroform formed at the bottom of the tube, was collected, evaporated to dryness under a nitrogen stream, and was subjected to the derivatization protocol with BSTFA with 1% TMCS. The derivatized mixture was evaporated to dryness and redissolved in *n*-hexane and injected into GC-MS.

### 3.4. Enrichment Factor and Extraction Recovery

To evaluate the performance of the extraction method, the enrichment factor (EF) and relative extraction recovery (ER%) were calculated using the following equations [3,31]:

(2) $$EF=\frac{C_{col}}{C_{aq}}$$

(3) $$ER\%=\frac{n_{col}}{n_{aq}}\times 100=\frac{C_{col}\times V_{col}}{C_{aq}\times V_{aq}}\times 100$$

where *C*_*col*_ is the analyte concentration in the collected organic phase; *C*_*aq*_ is the initial concentration of the analyte in the liquid sample; *n*_*col*_ is the total amount of analyte extracted in the collected organic phase; *n*_*aq*_ is the total amount of analyte in the liquid sample; and *V*_*col*_ and *V*_*aq*_ represent the volume of the collected organic phase and the volume of the liquid sample, respectively [31,38].

Following the Document SANTE 11312/2021 guidelines, the recovery must be within the range 70–120%, with an associated repeatability RSD ≤ 20% [42].

### 3.5. Statistical Approach Used for DLLME and SFOME Optimization

To achieve the best experimental conditions, a multivariate experimental design approach is considered in many cases [23,27,29].

Using statistical and mathematical criteria, the interaction effects resulting from the simultaneous variation in decisive factors and responses are observed, in order to maximize the extraction efficiency [27,43].

To realize the experimental design, first of all, the factors that influence the process are selected to produce the best experimental response. They are selected either from previous experience or from specialized literature. The factors are given levels, indicated by the numbers −1, 0, and +1 or other signs, depending on the software used. Level +1 is assigned to the factor that optimally meets the objective, –1 is assigned to the factor that does not meet the objective, and 0 is the average level of the factor [43].

The advantage of using a multivariate experimental design approach, if the factors are selected correctly, is saving time and money [43].

In the present work, a 2^3^ experimental design with a triplicate center point (0) was selected, with two levels (−1 and +1) for each factor. The factors studied were the amount of sodium chloride (*X*1), the volume of the dispersion solvent (acetonitrile, *X*2), and the volume of the extraction solvent (1-undecanol for SFOME and chloroform for DLLME, *X*3). The minimum, central, and maximum values assigned to the factors are shown in the following table (Table 6).

The desirability function was used to simultaneously optimize the experimental conditions to obtain the best recovery. It combines the individual responses into a composite function, where the factors decisively involved give the desired response [39].

The statistical software package JMP14 (SAS Institute, Cary, NC, USA) was used for statistical modeling.

### 3.6. Sample Collection

The wastewater sample was collected from the influent of the wastewater treatment plant from Cluj-Napoca, Romania, during a 24 h period. The composite sample was collected in a clean polypropylene flask, kept at 4 °C in the dark, transported to the laboratory, and stored in the freezer until analysis.

## 4. Conclusions

In the present research work, we propose fast, simple, and green SFOME and DLLME protocols for the simultaneous extraction and analysis of eight beta-blockers from wastewater samples, which were optimized and validated.

A 2^3^ experimental design with a triplicate center point (0) was used to optimize the extraction protocols, which reduced the number of experiments in the optimization steps.

A pertinent comparison between the advantages and disadvantages of the two chromatographic methods applied to the analysis of selected beta-blockers is provided.

The developed SFOME-LC-PDA protocol provides a satisfactory enrichment factor (60.14–88.16, except for atenolol (1.61) and nadolol (19.60)) and good extraction recovery (58.94–86.40%, except for atenolol (1.58%) and nadolol (19.20)) when the protocol was applied to the analysis of selected beta-blockers in wastewater samples.

For DLLME-GC-MS, the results obtained on wastewater provide an enrichment factor ranging from 190.03 to 246.38, except for atenolol, which was not detected, and nadolol, which has an EF of 11.85. The extraction recovery ranged from 63.34 to 82.13% for six of the eight compounds with the mention that atenolol was not detected, while for nadolol the recovery was 3.95%.

Although the SFOME-LC-PDA method is fast, both in terms of analysis time (18 min) and preparation of extracts for analysis (20 min), it has lower enrichment factors compared to the DLLME-GC-MS method.

The DLLME-GC-MS method, on the other hand, requires a longer analysis time (37 min), and in addition to the extraction time (20 min), it requires more than 1 h for derivatization of the compounds.

However, we can state that the methods developed by us show much better results compared to the experimental work carried out worldwide so far. The proposed microextraction protocols are easy to apply in any laboratory, use small volumes of sample and organic solvent, and have a short extraction time, being an appropriate and environmentally friendly alternative to traditional extraction techniques.

Even if the proposed protocols do not provide an adequate degree of extraction for all eight selected compounds, we can say that the information presented in this paper and the results obtained for the six compounds out of the eight selected in the study can constitute a starting point for the future optimization of other green extraction methods that can provide satisfactory results for all eight compounds.

## Figures and Tables

**Table 1:** Maximum, minimum, and optimum extraction recoveries obtained by simulation.

Beta-Blockers	Abbreviation	Extraction Recovery (%)					
		SFOME			DLLME		
		Minimum	Maximum	Optimum	Minimum	Maximum	Optimum
Atenolol	ATE	3.24	13.94	8.59	-	-	-
Nadolol	NAD	8.34	24.91	16.62	5.31	8.51	6.91
Pindolol	PIN	37.10	84.88	60.99	64.58	110.07	87.32
Acebutolol	ACE	26.25	73.03	49.64	−14.78	134.97	60.10
Metoprolol	MTP	40.14	94.59	67.36	98.82	161.62	130.22
Bisoprolol	BIS	45.56	119.46	82.51	99.69	181.64	69.94
Propranolol	PRP	67.42	136.69	102.05	106.36	151.00	128.68
Betaxolol	BTX	65.94	134.52	100.23	82.79	152.40	117.59

“-“ not identified.

**Table 2:** The performances of the SFOME-LC-PDA method.

Beta-Blockers	Calibration Curve Equation	SD	R^2^	LOD (µg/mL)	LOQ (µg/mL)	Precision (RSD%)		ER (%)	EF
						Intra-	Inter-		
ATE	y = 168,393x + 180,171	5764.51	0.9983	0.11	0.34	2.64	6.23	14.71	15.01
NAD	y = 119,820x + 194,465	2386.55	0.9991	0.07	0.20	2.91	2.37	29.85	30.46
PIN	y = 73,452x + 116,174	2877.82	0.9963	0.13	0.39	3.93	4.45	68.79	70.20
ACE	y = 72,148x + 22,066	2612.56	0.9998	0.12	0.36	1.23	2.37	59.99	61.22
MTP	y = 130,487x + 203,807	5902.37	0.9950	0.15	0.45	4.35	5.02	74.50	76.02
BIS	y = 130,628x + 142,398	5005.23	0.9974	0.13	0.38	0.73	2.73	81.45	83.11
PRP	y = 131,203x + 32,456	5322.12	0.9998	0.13	0.41	4.84	6.38	86.81	88.58
BTX	y = 127,756x + 107,522	4001.82	0.9981	0.10	0.31	4.87	6.10	87.55	89.34

**Table 3:** The performances of the DLLME-GC-MS method.

Derivatized Beta-Blockers	Calibration Curve Equation	SD	R^2^	LOD (µg/mL)	LOQ (µg/mL)	Precision (RSD%)		ER (%)	EF
						Intra-	Inter-		
ATE-2TMS	y = 341,497x + 79,567	27,525.77	0.9995	0.27	0.81	-	-	-	-
NAD–3TMS	y = 2E + 06x – 995,402	92,698.55	0.9999	0.15	0.46	7.38	10.74	3.14	11.79
PIN-2TMS	y = 567,017x + 191,180	118,922.34	0.9983	0.69	2.10	12.36	13.59	53.04	198.89
ACE-TMS	y = 219,605x – 332,187	8649.25	0.9999	0.13	0.39	8.33	11.08	56.89	212.75
MTP-TMS	y = 2E + 06x + 2E + 06	182,859.84	0.9984	0.30	0.91	10.34	11.17	78.91	220.91
BIS-TMS	y = 637,676x – 181,116	47,182.95	0.9997	0.24	0.74	12.60	14.26	92.1	243.97
PRP-TMS	y = 2E + 06x + 2E + 06	188,228.14	0.9984	0.31	0.94	8.82	11.48	71.6	212.75
BTX-TMS	y = 531,846x + 4113.8	35,621.76	0.9981	0.22	0.67	12.44	14.91	74.6	217.17

“-“ not estimated.

**Table 4:** The performances of the developed SFOME and DLLME protocol applied to wastewater.

Beta-Blockers	SFOME					DLLME				
	Spiked Amount (ng)	Wastewater		ER (%)	EF	Spiked Amount (ng)	Wastewater		ER (%)	EF
		Initial	Found				Initial	Found		
ATE	625	0.69	10.75	1.58	1.61	625	nd	nd	-	-
NAD		6.06	128.54	19.20	19.60		0.04	29.64	3.95	11.85
PIN		5.36	421.28	65.22	66.55		0.04	475.07	63.34	190.03
ACE		1.67	377.54	58.94	60.14		nd	494.67	65.96	197.87
MTP		117.87	554.90	68.53	69.92		1.28	599.56	79.94	239.82
BIS		17.14	550.40	83.61	85.32		nd	615.78	82.10	246.31
PRP		7.04	558.04	86.40	88.16		0.20	540.56	72.07	216.22
BTX		17.03	553.64	84.14	85.86		nd	615.94	82.13	246.38

“nd” not detected; “-“ not calculated.

**Table 5:** Comparison between the proposed SFOME and DLLME with other microextractions of beta-blockers.

Beta-Blockers	Method	Matrix	Sample Volume (mL)	Extraction Solvent (mL)	LOQ (µg/mL)	ER (%)	EF	Ref.
metoprolol, propranolol, carvedilol, diltiazem, verapamil	DLLME	plasma	0.66	100 µL dichloromethane	0.007–0.019	4.4–10.8	33–82	[3]
racemic propranolol	DLLME-SFO	plasma	0.5	40 µL of 1-undecanol	0.05	13.3–13.0	16.7–16.2	[23]
metoprolol, propranolol	TDLLME	plasma, wastewater	10	103 µL of 1,2-dichloroethane and 45 µL of aqueous extractant phase with pH value of 2.0	0.028–0.003	34–45	75–100	[27]
atenolol, acebutolol, bisoprolol, betaxolol	VA DLPME-SFOD	river water, wastewater	5	50 µL of dodecanol	0.01–0.07	-	23–55	[29]
carvedilol	DLLME-SFO	plasma	1	75 µL of 1-undecanol	0.04 ^1^	-	-	[34]
atenolol, propranolol, carvedilol	AALLME-SFOD	urine	1	125 µL of 1-dodecanol and 160 µL of water with 1000 mg/L Triton X-100	0.26–0.30	64.22–94.57	11.24–16.55	[35]
atenolol, nadolol, pindolol, acebutolol, metoprolol, bisoprolol, propranolol, betaxolol	SFOME	wastewater	10	100 µL of 1-undecanol	0.20–0.45	58.94–86.40	60.92–88.16	This study.
	DLLME			75 µL chloroform	0.39–1.23	63.34–82.13	190.03–246.38	

^1^ Lower limit of quantification. “-” no data available.

**Table 6:** Minimum, central, and maximum values of the factors used to create experimental design.

Factors	Values for DLLME and SFOME		
	Lowest (–1)	Central Point (0)	Highest (+1)
NaCl (g) (*X*1)	0.5	1.25	2
Disperser (µL) (*X*2)	100	175	250
Extractant (µL) (*X*3)	50	75	100

## Data Availability

Data are contained within the article and Supplementary Materials.

## Supplementary Materials

The following supporting information can be downloaded at: https://www.mdpi.com/article/10.3390/molecules30051016/s1. Table S1: The set code value of factors used in each SFOME experiment and the values of extraction recovery (%) obtained for each of the studied pharmaceuticals; Table S2: The set code value of factors used in each DLLME experiment and the values of extraction recovery (%) obtained for each of the studied pharmaceuticals;.Table S3: The experimental conditions of each protocol of experiments; Table S4: The magnitude of the effects and the significance level for the factors and their interactions for the SFOME protocol; Table S5: The magnitude of the effects and the significance level for the factors and their interactions for the DLLME protocol; Table S6: The molecular structure and some physico-chemical properties of the studied pharmaceuticals; Figure S1: The optimal conditions predicted by model, minimum, maximum and optimum extraction recovery values, and the value of desirability function for the SFOME protocol; Figure S2: The optimal conditions predicted by model, minimum, maximum and optimum extraction recovery values, and the value of desirability function for the DLLME protocol.

## Abbreviations

The following abbreviations are used in this manuscript:

DLLME	Dispersive Liquid–Liquid Microextraction
SFOME	Solidification Of Floating Organic Droplet Microextraction
GC	Gas Chromatography
LC	Liquid Chromatography
GC–MS	Gas Chromatography coupled with Mass Spectrometry
HPLC–PDA	High-Performance Liquid Chromatography with Photodiode Array Detector
LOQ	Limit of Detection
LOD	Limit of Quantification
SPE	Solid-Phase Extraction
SPME	Solid-Phase Microextraction
SBSE	Stir-Bar Sorptive Extraction
FPSE	Fabric-Phase Sorbent Extraction
µQuEChERS	Micro Quick, Easy, Cheap, Effective, Rugged, and Safe
SDME	Single-Drop Microextraction
HF-LPME	Hollow-Fiber Liquid-Phase Microextraction
ATE	Atenolol
NAD	Nadolol
PIN	Pindolol
ACE	Acebutolol
MET	Metoprolol
BIS	Bisoprolol
PRP	Propranolol
BTX	Betaxolol
RSD	Relative Standard Deviation
SD	Standard Deviation
S	the Slope of each the Calibration Curve
ICH	International Committee for Harmonization
EF	Enrichment Factor
ER	Enrichment Recovery
BSTFA	N,O-bis(trimethylsilyl)trifluoroacetamide
TMCS	Trimethylchlorosilane
TMS	trimethylsilyl
DLLME-SFO	Dispersive Liquid–Liquid Microextraction based on Solidification pf Floating Organic Droplet
VA DLPME-SFOD	Vortex Assisted Dispersive Liquid-Phase Microextraction with Solidification of Floating Organic Droplet
TDLLME	Tandem Dispersive Liquid–Liquid Microextraction
AALLME-SFOD	Air-Assisted Liquid–Liquid Microextraction with Solidification of Floating Organic Droplet
ACN	Acetonitrile
MS	Mass Spectrometry
PDA	Photodiode Array Detector
USA	United States of America
