# Supplementary material for: The Effectiveness of Liquid-Phase Microextraction of Beta-Blockers from Aqueous Matrices for Their Analysis by Chromatographic Techniques
Source: Molecules. 2025 Feb 22;30(5):1016. doi: 10.3390/molecules30051016 (PMC11901778; doi:10.3390/molecules30051016)

SUPPLEMENTARY INFORMATION

Article

The Effectiveness of Liquid-Phase Microextraction of Beta-Blockers from Aqueous Matrices for Their Analysis by Chromatographic Techniques

Mihail Simion Beldean-Galea [1,2*], Mihaela-Cătălina Herghelegiu [1,2*], Vlad-Alexandru Pănescu [1,2], Jérôme Vial[3], Maria Concetta Bruzzoniti[4], Maria-Virginia Coman [2]

[1]-Faculty of Environmental Science and Engineering, Babeș-Bolyai University, 30 Fântânele Str., RO-400294 Cluj-Napoca, Romania;
[2] "Raluca Ripan" Institute for Research in Chemistry, Babeş-Bolyai University, 30 Fântânele Str., RO-400294 Cluj-Napoca, Romania;
[3] Chemistry, Biology and Innovation Department, École Supérieure de Physique et de Chimie Industrielles ESPCI Paris PSL, 10 Rue Vauquelin, 75005 Paris, France
[4] Department of Chemistry, University of Turin, Via P. Giuria 5, 10125 Turin, Italy
* Correspondence: simion.beldean@ubbcluj.ro, mihaela.herghelegiu@ubbcluj.ro

The supplementary information contains 10 pages, and includes 6 tables and 2 figures.

List of contents of the supplementary information:

Table S1. The set code value of factors used in each SFOME experiment and the values of extraction recovery (%) obtained for each of the studied pharmaceuticals

Table S2. The set code value of factors used in each DLLME experiment and the values of extraction recovery (%) obtained for each of the studied pharmaceuticals

Tabel S3. The experimental conditions of each protocol of experiments

Table S4. The magnitude of the effects and the significance level for the factors and their interactions for the SFOME protocol

Table S5. The magnitude of the effects and the significance level for the factors and their interactions for the DLLME protocol

Table S6. The molecular structure and some physico-chemical properties of the studied pharmaceuticals

Figure S1. The optimal conditions predicted by model, minimum, maximum and optimum extraction recovery values, and the value of desirability function for the SFOME protocol.

Figure S2. The optimal conditions predicted by model, minimum, maximum and optimum extraction recovery values, and the value of desirability function for the DLLME protocol.

Table S1. The set code value of factors used in each SFOME experiment and the values of extraction recovery (%) obtained for each of the studied pharmaceuticals

Exp. no.	Salt (X1)	Disperser (X2)	Extractant (X3)	Atenolol	Acebutolol	Nadolol	Pindolol	Metoprolol	Bisoprolol	Propranolol	Betaxolol
1.	-1	-1	-1	0	0.16	0	0.31	0.92	1.12	3.49	2.15
2.	-1	-1	1	0	0.17	0	0.71	0.83	1.57	6.85	3.25
3.	-1	1	-1	3.34	44.6	0.21	0.49	1.49	4.11	20.16	18.49
4.	-1	1	1	0	1.12	0.19	1.6	3.09	0.83	36.32	28.3
5.	1	-1	-1	0.44	12.06	2.67	19.46	21.52	32.12	72.91	63.28
6.	1	-1	1	3.01	23.95	7.63	34.53	37.62	51.27	85.03	79.33
7.	1	1	-1	2.89	41.38	6.15	48.89	55.01	67.58	71.72	72.7
8.	1	1	1	14.47	60.44	24.81	68.55	74.11	85.94	89.42	91.16
9.	0	0	0	2.67	32.68	8.53	40.78	47.47	64.48	81.13	78.52
10.	0	0	0	1.76	34.3	7.82	43.43	49.45	67.65	84.39	81.91
11.	0	0	0	3.33	33.36	7.31	42.18	48.74	68.52	88.61	85.24

Table S2. The set code value of factors used in each DLLME experiment and the values of extraction recovery (%) obtained for each of the studied pharmaceuticals

Exp. no.	Salt (X1)	Disperser (X2)	Extractant (X3)	Atenolol	Acebutolol	Nadolol	Pindolol	Metoprolol	Bisoprolol	Propranolol	Betaxolol
1.	-1	-1	-1	0	0	0	0	0	0	0	0
2.	-1	-1	1	0	0	0	0	0	0	0	0
3.	-1	1	-1	0	0	0	0.35	31.37	23.87	22.26	18.07
4.	-1	1	1	0	0	0	0.05	2.15	0	0.24	0
5.	1	-1	-1	0	0	0.48	4.3	7.94	19.48	4.37	15.79
6.	1	-1	1	0	0	0	0.09	5.51	2.68	0.88	3.83
7.	1	1	-1	0	59.63	7.4	82.05	118.81	144.39	121.39	116.65
8.	1	1	1	0	50.63	6.84	89.63	150.24	131.43	140.61	112.21
9.	0	0	0	0	22.43	1.06	23.92	16.34	39.85	20.01	37.08
10.	0	0	0	0	73.54	1.53	40.36	26.41	74.02	25.65	62.7
11.	0	0	0	0	0	0.66	18.22	28.43	37.19	37.47	34.96

Tabel S3. The experimental conditions of each protocol of experiments

Exp. no.	Salt (X1) NaCl (g)	Disperser (X2) ACN (µL)	Extractant (X3) 1-undecanol/chloroform (µL)
1.	0.5	100	50
2.	0.5	250	50
3.	0.5	100	100
4.	0.5	250	100
5.	2	100	50
6.	2	250	50
7.	2	100	100
8.	2	250	100
9.	1.25	175	75
10.	1.25	175	75
11.	1.25	175	75

Table S4. The magnitude of the effects and the significance level for the factors and their interactions for the SFOME protocol

Source	Logworth	PValue	
Salt*Extractant	2.442	0.00362	
Salt	2.440	0.00363	^
Disperser	2.424	0.00376	
Salt*Disperser*Extractant	2.010	0.00978	
Extractant	1.849	0.01417	^
Salt*Disperser	1.822	0.01507	^
Disperser*Extractant	1.133	0.07361	^

Table S5. The magnitude of the effects and the significance level for the factors and their interactions for the DLLME protocol

Source	Logworth	PValue
Disperser	2.621	0.00239
Salt	2.454	0.00351
Salt*Disperser	2.399	0.00399
Salt*Disperser*Extractant	0.656	0.22056
Salt*Extractant	0.603	0.24965 ^
Extractant	0.410	0.38882 ^
Disperser*Extractant	0.140	0.72393 ^

Table S6. The molecular structure and some physico-chemical properties of the studied pharmaceuticals

**Beta-blocker**	**Molecular structure**	**Chemical formula**	**Molecular weight (g/mol)**	**LogP**	**pKa**
**Atenolol**	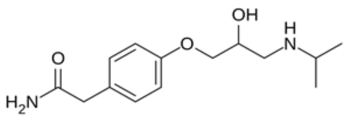	$C_{14}H_{22}N_2O_3$	266.34	0.16	9.58
**Nadolol**	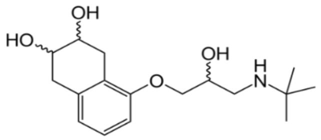	$C_{17}H_{27}NO_4$	309.4	0.81	9.67
**Pindolol**	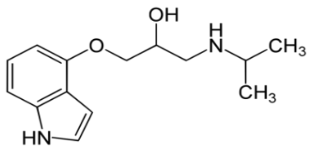	$C_{14}H_{20}N_2O_2$	248.32	1.75	9.54
**Acebutolol**	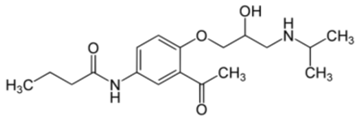	$C_{18}H_{28}N_2O_4$	336.40	1.71	9.52
**Propranolol**	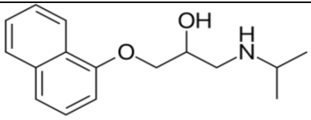	$C_{16}H_{21}NO_2$	259.34	3.48	9.53
**Bisoprolol**	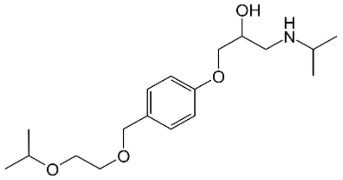	$C_{18}H_{31}NO_4$	325.40	2.20	9.57
**Betaxolol**	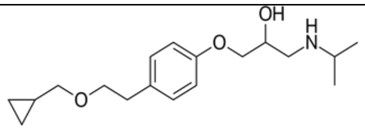	$C_{18}H_{29}NO_3$	307.40	2.81	9.21
**Metoprolol**	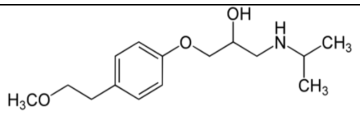	$C_{15}H_{25}NO_3$	267.36	2.15	9.56

Physicochemical properties (Molecular weight, LogP, pKa) from PubChem databases. https://pubchem.ncbi.nlm.nih.gov/ (Accessed November 2024).

Figure S1. The optimal conditions predicted by model, minimum, maximum and optimum extraction recovery values, and the value of desirability function for the SFOME protocol.

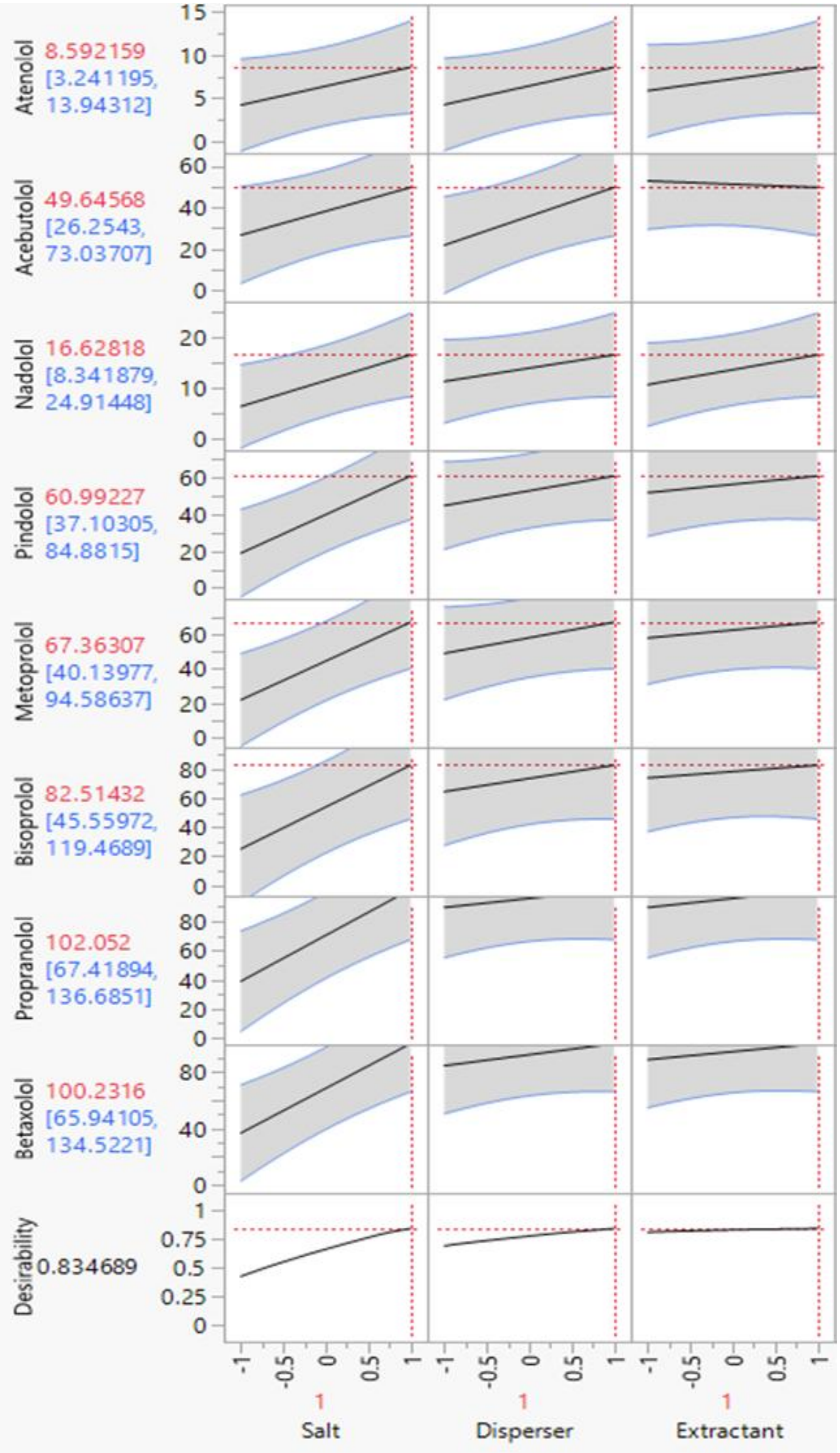

Figure S2. The optimal conditions predicted by model, minimum, maximum and optimum extraction recovery values, and the value of desirability function for the DLLME protocol.

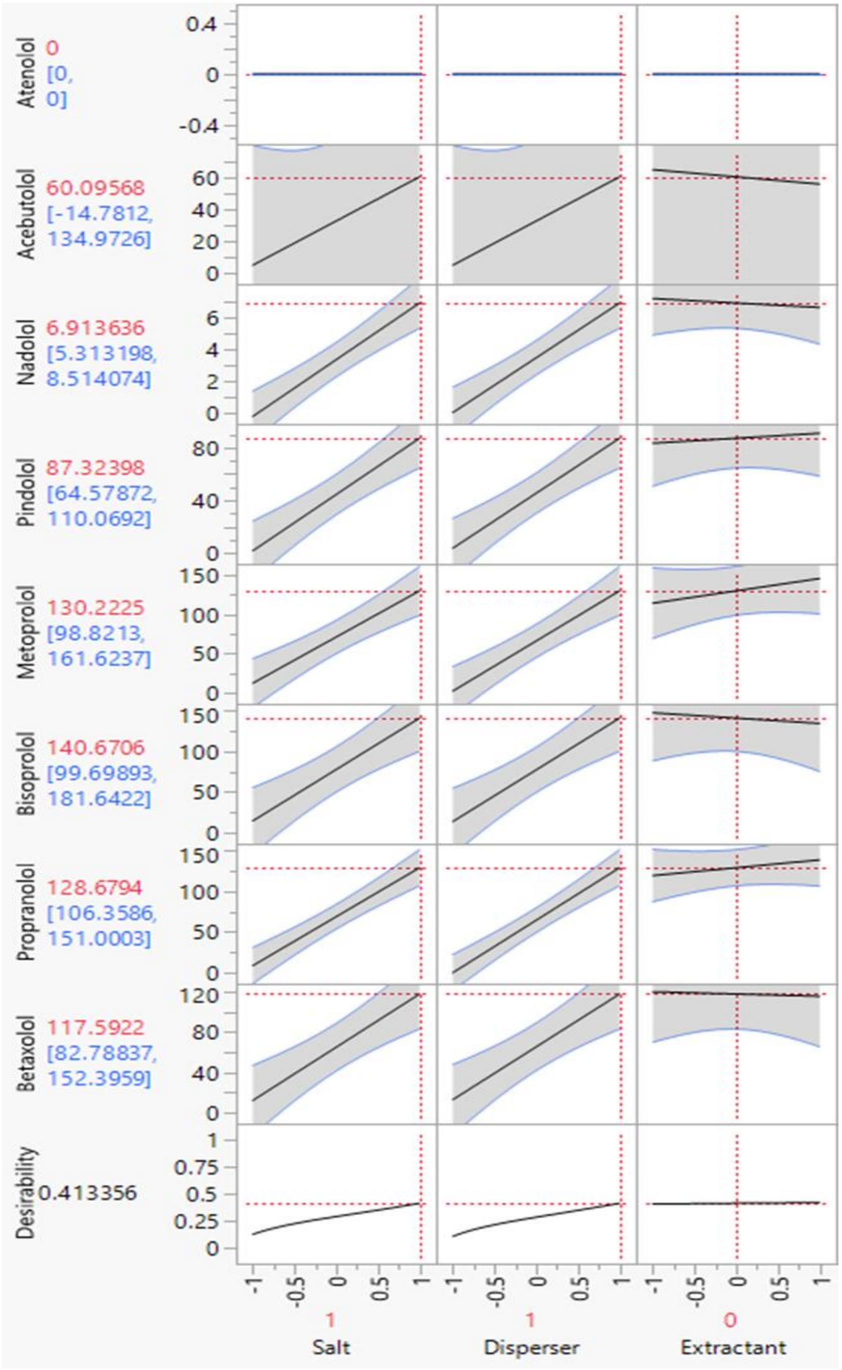

Supplement: Supplementary file 1 [file molecules-30-01016-s001.zip › molecules-3471098-supplementary.pdf]
